# Impact of socioeconomic position on the utilization of rehabilitation services among the Chinese older adults disabled by injury

**DOI:** 10.3389/fpubh.2023.1034482

**Published:** 2023-03-21

**Authors:** Yi-Ran Wang, Ya-Nan Luo, Ya-Lin Fan, Yun-Yi Fan, Xiao-Ying Zheng

**Affiliations:** ^1^APEC Health Science Academy (HeSAY), PKU, Beijing, China; ^2^School of Population Medicine and Public Health, Chinese Academy of Medical Sciences/Peking Union Medical College, Beijing, China; ^3^Department of Global Health, School of Public Health, Peking University, Beijing, China; ^4^School of Public Health, Capital Medical University, Beijing, China

**Keywords:** aging, disability, rehabilitation service, socioeconomic position, injury

## Abstract

**Aim:**

This study aimed to explore the utilization of rehabilitation services and associated socioeconomic position (SEP) factors among Chinese older adults disabled by injury.

**Methods:**

Data from the second China National Sample Survey on Disability (CSSD) were used in this study. Chi-square test was used to analyze the significant differences between groups, and binary logistic regression model was used to calculate the odds ratios and 95% confidence intervals for socioeconomic factors associated with utilization of rehabilitation services among Chinese older adults disabled by injury.

**Results:**

Among the older adults disabled by injury in the CSSD, the gap between demand and utilization of medical treatment, assistive devices and rehabilitation training were around 38, 75, and 64%, respectively. This study revealed two relationship patterns (“high-low-high” and “low-high-low”) among SEP, prevalence of injury-caused disability and odds of utilization of rehabilitation services among the Chinese older adults disabled by injury, that is, the older adult with higher SEP have a lower prevalence of injury-caused disability, but a higher odds of utilization of rehabilitation services; conversely, the older adults with lower SEP have a relatively higher prevalence but a lower odds of utilization of rehabilitation services.

**Conclusion:**

There is a large gap between the high demand and low utilization of rehabilitation services among the Chinese older adults disabled by injury, especially for those living in the central or western regions or rural areas, without insurance or disability certificate, having the annual household per capita income lower than the national average or lower educational level. Strategies to improve the disability manage system, to strengthen the chain of “information discovery-information transmission-rehabilitation services supply-continuous health monitoring and management” for the older adults disabled by injury are warranted. In view of the poor and illiterate groups among the disabled older adults, to enhance medical aids and popularize the scientific information to compensate for the lack of affordability and awareness of rehabilitation services utilization is essential. In addition, it is necessary to further expand the coverage and improve the payment system of medical insurance for rehabilitation services.

## Introduction

Injury has become the main cause of death and disability in most countries (1), accounting for 8% of the global causes of death (2). Temporary or permanent disability by injury contributes to an even greater disease burden (3). In China, injury has exceeded infectious diseases and ranked the second cause of the disease burden. Every year, there are 700,000–800,000 deaths and 62 million emergency visits due to injury (4). With the growth of population life expectancy in China, the percentage of life expectancy with disability is also continuously expanding, and is expected to increase from 9.60% in 2015 to 14.54% in 2050 (5). The problem of “disability expansion” is more severe among the older adults in China, which is estimated to experience 5.78 years lived with disability in 2015, and this number is expected to increase to 11.45 in 2050 (5). The years lived with disability (YLD) caused by injury among the older adults over 70 years old has increased year by year since 2005 (6).

Rehabilitation service needs are universal, a conclusion confirmed by a study based on the Global Burden of Disease (GBD) 2019, which estimates that in 2019, 2.41 billion individuals had conditions that would benefit from rehabilitation, that is, at least one in every three people in the world needs rehabilitation at some point in the course of their illness or injury (7). However, the utilization of rehabilitation services is disproportionately insufficient (8, 9). As for the injury-caused disabled, a Chinese study revealed the challenge of significant under-utilization of rehabilitation services (10).

Based on this, some studies have explored more possible strategies to remove the barriers to the utilization of rehabilitation services for the disabled from the perspective of the socioeconomic position (SEP). And yet, to the best of our knowledge, there may be few studies on rehabilitation services and associated SEP factors for the Chinese older adults disabled by injury. Taken all these into consideration, this article analyzes the utilization of rehabilitation services and related SEP factors for the Chinese older adults with injury-caused disability. In this way, it aims to supplement the research in this field and provide empirical data in making rehabilitation policies for the older adults disabled by injury in China.

## Methods

### Data source

In this study, the Second National Sampling Survey on Disability in China in 2006 (CSSD) database was used to extract samples of the older adults aged 60 and above who were disabled by injury. It adopts the stratified, multi-stage and cluster probability proportional sampling method. It covers a total of 2,526,145 people from 771,797 households in 31 provinces, autonomous regions, and cities, with a sampling ratio of 1.93‰. The survey data is authentic (11).

### Core concepts

#### Disability

Disability refers to the abnormality or the loss of anatomical structure and physiological function caused by various physical and mental diseases, or injuries, or congenital abnormalities, which results in long-term, continuous or permanent dysfunction of the body that cannot be cured (12).

#### Injury

Injury is defined as tissue damage or hypoxia by asphyxia that affect individual activities and need treatment or rehabilitation, resulting from energy transfer or interference exceeding the limits of biological endurance (13).

#### Injury-caused disability

Injury-caused disability is defined as a person who completely or partially loses the ability to engage in certain activities normally due to the loss or abnormality of certain organization or function in physiology and psychology caused by injury (10).

#### Medical rehabilitation

As an important part of rehabilitation, medical rehabilitation is the application of rehabilitation concept in the medical field. It refers to healing the dysfunction of the sick, the injured, and the disabled through medical means (14). The rehabilitation referred to in CSSD is medical rehabilitation, including medical treatment, assistive devices and rehabilitation training.

#### Socioeconomic position

Socioeconomic position (SEP) is an economic and sociological combined total measure of an individual's or family's economic access to resources and social position in relation to others (15, 16). When analyzing SEP, three variables, namely, income, education, and occupation are examined (17).

### Study design and subjects

This research aims to study the demand and utilization of rehabilitation services in the Chinese older adults aged 60 and above who are disabled by injury, and the correlation between the utilization of rehabilitation services and individual's SEP. In the CSSD, it lists only organic and non-organic mental disorders as the causes of mental disability based on ICD-10 diagnostic criteria, failing to examine the injury-related causes (18, 19). Therefore, this research focuses on the older adults with vision, hearing, speech, physical, and intellectual disabilities. Following the existing research (20, 21), this paper extracts the injury-related causes of disability from the CSSD as follows (Table 1):

**Table 1:** Injury-related causes of disability in the CSSD.

**Disability type**	**Causes of disability**
Vision	Trauma, poisoning
Hearing	Drug poisoning, trauma or accidental injury, noise, detonation
Speech	Brain injury, birth injury, CO poisoning
Physical	Industrial injury, traffic accident, spinal cord injury, brain injury, other injuries, poisoning
Intellectual	Birth injury, industrial injury, traffic accident, other injuries, poisoning, allergic reaction

In this study, the dependent variable is “the usage of rehabilitation services”, that is, as long as any one of “medical services and assistance, assistive devices, rehabilitation training and services” is used, the participant is considered to have used rehabilitation services. Since the older adult/adults are less affected by occupation, the explanatory variables of SEP include annual household per capita income and educational level. The higher the annual household per capita income or educational level, the higher the SEP. In the descriptive analysis, the annual disposable per capita income of urban residents in China in 2006 was 11,759 yuan, and the per capita net income of rural residents was 3,587 yuan, according to the data of the National Bureau of Statistics. In this study, the annual per capita income of urban households that is higher than 11,759 yuan and the per capita net income of rural households that is higher 3,587 yuan are defined as higher than the annual per capita income of national residents (22). In addition, according to regulation of the National Bureau of Statistics, the Eastern China includes Beijing, Tianjin, Hebei, Shanghai, Jiangsu, Zhejiang, Fujian, Shandong, Guangdong and Hainan; the Central China includes Shanxi, Anhui, Jiangxi, Henan, Hubei and Hunan; the Western China includes Inner Mongolia, Guangxi, Chongqing, Sichuan, Guizhou, Yunnan, Tibet, Shaanxi, Gansu, Qinghai, Ningxia and Xinjiang; and the Northeastern China includes Liaoning, Jilin, and Heilongjiang (23).

### Ethical approval

These surveys were approved by the State Council [Guo Ban Fa No. 77 (1986); Guo Ban Fa No. 73 (2004)] and the survey plan was formulated in accordance with the Statistics Law of the People's Republic of China and the Law of the People's Republic of China on the Protection of Disabled Persons. During the surveys, all respondents provided informed consent to participate in the surveys and clinical diagnosis.

### Statistical analysis

Stata 16.0 was used for data analysis. Chi square test was employed for univariate analysis, and binary logistic regression was adopted for multivariate analysis. Hosmer-Lemeshow test was used for the test of goodness of fit of the model. The significance level was 0.05.

## Results

### Prevalence of injury-caused disability in the older adults

A total of 354,859 elders were surveyed in CSSD, including 85,260 disabled elders. Among them, 10,314 older adults people were disabled by injury, with the prevalence of 291/10,000 (number of the disabled older adults by injury in CSSD/total number of the older adults in CSSD), accounting for 12.1% of all older adults disabled people. The prevalence of physical disability by injury is the highest in this group (151/10,000) (number of the older adults with physical disabilities caused by injury and without other kinds of disabilities in CSSD/total number of the older adults in CSSD), followed by hearing disability (71/10,000). On the contrary, the prevalence is relatively low for the visual (12/10,000), intellectual (2/10,000), and speech (0.4/10,000) disabilities caused by injury in the older adults. As for the disability grading, most injury-caused disability in the older adult is mild (grade IV), accounting for nearly 57% of the total, followed by moderate (grade III, nearly 28%). Severe (grade II) and extremely severe (grade I) disabled elders account for only 15%.

In terms of SEP factors, the injury-caused disability in the older adults shows a higher prevalence in the groups of those with the annual household per capita income lower than the national average (prevalence = 306/10,000, reference: those with the annual household per capita income higher than the national average, prevalence = 224/10,000), the illiteracy/with no schooling (prevalence = 321/10,000, reference: primary school or above, reference = 267/10,000) (Table 2).

**Table 2:** Comparison of the prevalence of the disabled older adults by injury with different SEP.

	**Total**	**Disabled by injury**	**Prevalence/10,000**	**χ^2^**	***P***
**Annual household per capita income**					
≥National average	67,922	1,523	224	131.3268	<0.05
<National average	286,937	8,791	306		
**Educational level**					
Illiteracy/no schooling	158,014	5,066	321	90.5672	<0.05
Primary school or above	196,845	5,248	267		

### The demand and utilization of rehabilitation services of the older adults with injury-related disability

There is a large gap between the high demand and low utilization of rehabilitation services among the Chinese older adults disabled by injury (hereinafter referred to as the “rehabilitation gap”). Thirty-eight percentage of demand for medical treatment, 75% for assistive devices and 64% for rehabilitation training are left unsatisfied. Based on social and demographic factors, there is little difference in the rehabilitation gap among different disability grades, gender, age groups, and marital status. However, the rehabilitation gap is relatively larger among the older adults with injury-related disability who (1) live in the central or western regions, (2) are rural residents, (3) without insurance, (4) without disability certificate, (5) have the annual household per capita income lower than the national average, and (6) are illiterate or with no schooling (Table 3).

**Table 3:** Demand and utilization of rehabilitation services of the older adults with injury-related disability.

	**Medical services and assistance**			**Assistive devices**			**Rehabilitation training and services**		
	**Demand (** ***n*** **)**	**Usage (** ***n*** **)**	**^#^ Gap (%)**	**Demand (** ***n*** **)**	**Usage (** ***n*** **)**	**Gap (%)**	**Demand (** ***n*** **)**	**Usage (** ***n*** **)**	**Gap (%)**
Overview	7,191	4,500	37.42	4,983	1,251	74.89	2,952	1,086	63.21
**Disability grade**									
Mild (grade IV)	4,121	2,611	36.64%	2,438	621	74.53%	1,794	666	62.88%
Moderate to extremely severe (grade I–III)	3,070	1,889	38.47%	2,545	630	75.25%	1,158	420	63.73%
**Gender**									
Male	4,011	2,616	34.78	2,917	741	74.60	1,616	621	61.57
Female	3,180	1,884	40.75	2,066	510	75.31	1,336	465	65.19
**Age groups**									
60–69	2,703	1,669	38.25%	1,677	405	75.85%	1,150	410	64.35%
70–79	3,012	1,901	36.89%	2,185	554	74.65%	1,196	464	61.20%
≥1.	1,476	930	36.99%	1,121	292	73.95%	606	212	65.02%
**Marital status**									
Without spouse	2,827	1,641	41.95%	1,970	468	76.24%	1,156	397	65.66%
Having spouse	4,364	2,859	34.49%	3,013	783	74.01%	1,796	689	61.64%
**Region**									
Western China	2,563	1,454	43.27	1,736	345	80.13	1,131	302	73.30
Eastern China	2,377	1,822	23.35	1,802	566	68.59	971	455	53.14
Central China	1,755	803	54.25	1,092	219	79.95	592	187	68.41
Northeastern China	496	421	15.12	353	121	65.72	258	142	44.96
**Residence**									
Rural	4,872	2,688	44.83	3,257	627	80.75	1,886	587	68.88
Urban	2,319	1,812	21.86	1,726	624	63.85	1,066	499	53.19
**Insurance (including endowment, medical, insurance, employment injury, and unemployment insurance)**									
No	4,364	2,424	44.45%	2,934	693	76.38%	1,762	608	65.49%
Yes	2,827	2,076	26.57%	2,049	558	72.77%	1,190	478	59.83%
**Disability certificate**									
No	6,108	3,656	40.14%	4,321	992	77.04%	2,388	845	64.61%
Yes	1,083	844	22.07%	662	259	60.88%	564	241	57.27%
**Annual household per capita income**									
<National average	6,216	3,740	39.83	4,174	988	76.33	2,524	913	63.83
≥National average	975	760	22.05	809	263	67.49	428	173	59.58
**Educational level**									
Illiteracy/no schooling	3,634	2,008	44.74	2,397	499	79.18	1,405	454	67.69
Primary school or above	2,416	1,566	35.18	1,701	427	74.90	1,008	391	61.21
**Chi-square test (the variables are demographic characteristics and whether they use rehabilitation services or not. Inside the brackets is** ***p*****-value)**									
Gender	5.9398 (<0.05)			28.6559 (<0.05)			10.7134 (<0.05)		
Age groups	1.5856 (=0.208)			1.8787 (=0.170)			0.0321 (=0.858)		
Marital status	0.9589 (=0.619)			15.4828 (<0.05)			1.5392 (=0.463)		
Region	25.5373 (<0.05)			1.9741 (=0.160)			3.6353 (=0.057)		
Residence	304.0136 (<0.05)			116.2728 (<0.05)			134.4611 (<0.05)		
Insurance	200.2527 (<0.05)			187.7017 (<0.05)			94.7389 (<0.05)		
Disability certificate	101.8897 (<0.05)			9.5350 (<0.05)			5.9190 (<0.05)		
Annual household per capita income	54.8969 (<0.05)			26.4949 (<0.05)			38.1499 (<0.05)		
Educational level	28.5757 (<0.05)			44.2832 (<0.05)			1.3060 (=0.253)		

# The calculation of the rehabilitation gap: (1 – the number of people using/demanding the service) ^*^100%.

^*^Indicates a significant difference, *p* < 0.05.

### Associated SEP factors of rehabilitation service utilization among the older adults with disability caused by injury

Binary logistic regression analysis was carried out with “the usage of rehabilitation services” as the dependent variable; and disability grade, gender, age, marital status, region, residence (rural/urban), insurance status, and disability certificate status as the control variables; and annual household per capita income, educational level as the explanatory variables. The assignment of independent variables is shown in Table 4. Hosmer-Lemeshow goodness of fit test result shows that Hosmer-Lemeshow χ^2^ = 12.77, *P* = 0.12 > 0.05, which means that the model fits better.

**Table 4:** Variables assignment.

**Variables**	**Assignment**
**Dependent variables**	
Usage of rehabilitation services	0 = Never used, 1 = have used
**Control variables**	
Disability grade	1 = Grade I, 2 = grade II, 3 = grade III, 4 = grade IV
Gender	0 = Female, 1 = male
Age	Continuous variable
Marital status	0 = Without spouse, 1 = having spouse
Region	0 = Western China, 1 = eastern China, 2 = central China, 3 = northeastern China
Residence	0 = Rural areas, 1 = urban areas
Insurance status	0 = No, 1 = yes
Disability certificate status	0 = No, 1 = yes
**Explanatory variables**	
Annual household per capita income	0 = Lower than the national average, 1 = Higher than the national average
Educational level	0 = Illiteracy/no schooling, 1 = Primary school or above

The results show that after controlling the variables of disability grade, gender, age, marital status, region, residence (rural/urban), insurance status, and disability certificate status, the odds of utilization of rehabilitation services is much higher in the groups of participants that (1) with higher annual household per capita income (OR = 1.184, 95%CI 1.054–1.329, reference: annual household per capita income < national average), (2) have been to primary school or above (OR = 1.143, 95%CI 1.038–1.256, reference: illiteracy/no schooling) (Table 5).

**Table 5:** Regression analysis of the SEP factors on whether the older adults disabled by injury utilize the rehabilitation services.

**The usage of rehabilitation services**	***B***	**Robust S.E**.	**OR (95% CI)**	***P***
**Annual household per capita income (reference: <national average)**				
≥National average	0.169	0.070	1.184 (1.054–1.329)	<0.05
**Educational level (reference: illiteracy/no schooling)**				
Primary school or above	0.132	0.056	1.142 (1.038–1.256)	<0.05

OR, odds ratio; CI, confidence intervals.

## Discussion

The gap between the utilization of rehabilitation services and the demand is large for the older adults disabled by injury. Rehabilitation services needs are universal (7). As for the older adults, their needs for rehabilitation services are neglected more serious (24, 25). There are few studies on the utilization of rehabilitation services for the older adult/adults disabled by injury in China. An similar study based on CSSD shows that the rehabilitation gap for the older adults disabled by injury is nearly 40% (26). This study pointed out the problem of low utilization rate of rehabilitation services from the perspective of all the disabled by injury. In addition, this study revealed two relationship patterns (“high-low-high” and “low-high-low”) among SEP, prevalence of injury-caused disability and odds of utilization of rehabilitation services among the Chinese older adults disabled by injury, that is, the older adults with higher SEP have a lower prevalence of injury-caused disability, but a higher odds of utilization of rehabilitation services; conversely, the older adults with lower SEP have a relatively higher prevalence but a lower odds of utilization of rehabilitation services.

Existing research showed a “inverse combination” pattern between individual health status and SEP (27), that is, the lower the SEP, the worse the individual health status (28). This explains the inverse combination of the SEP and the prevalence of disability by injury among the older adults in this study. educational level (29–31) and income (32, 33) are two important factors affecting the utilization of health services, and the mechanism by which they affect the individual behaviors mainly are the two intermediate factors of “intention” and “affordability”. The essence of individual utilization of health services is consumption behavior. Based on the economic theory, “intention” and “affordability” are the two determinants of consumption behavior. Some foundation theories have emphasized the important effects of income and education on individual health services utilization behavior. Anderson health services utilization behavior model points out that “intention” comes from the individual awareness of the gap between their actual health level and the ideal state or the diagnosis from a medical professional, as well as the individual's analysis of the benefits of the health behaviors, which involves the individual's health literacy affected by the educational level deeply (34). Affordability is mainly determined by individual “ability resources”, that is, the ability of individuals to obtain health service resources, in which income is the basic composition (34). There is also an important psychological variable between “intention” and “affordability”. Social psychologist Icek Ajzen believes that “perceived behavioral control” is an important intermediary in the transformation path from intention to action (35). Through the judgment of affordability, individual clearly assesses the difficulty of taking practical actions, and then decides whether to adopt healthy behaviors. In addition, the social ecology model also reveals that individual behavior is determined by individual ability, knowledge, motivation, etc. (36).

## Limitations

However, there are some limitations that require consideration. First, It fails to distinguish mentally disabled persons due to injury in the CSSD database. Therefore, this study lacks the situation of mentally disabled people. Second, The CSSD database is relatively old and has limited practical guidance. Nonetheless, it is still the latest national sampling survey on disability in China.

## Conclusions

Facing the current situation of limited rehabilitation services in China, it is an essential means to improve the efficiency of rehabilitation services by clarifying the vulnerable groups of people with injury-caused disability and taking precise assistance measures. To be more specific, according to the distribution of the prevalence of injury-related disability and the utilization rate of rehabilitation services among different older adults groups, targeted measures should be taken based on their specific characteristics, especially the rural residents or people living in the central or western regions, poverty-stricken people, people with low educational level, or without insurance and disability certificate among the older adults. Strategies to improve the disability manage system, to strengthen the chain of “information discovery-information transmission-rehabilitation services supply-continuous health monitoring and management” for the older adults with injury-caused disability are warranted, especially for the vulnerable groups aforementioned. In view of the poor and illiterate groups, to enhance medical aids and popularize the scientific information to compensate for the lack of affordability and awareness of rehabilitation services utilization is essential. In addition, it is necessary to further expand the coverage and improve the payment system of medical insurance for rehabilitation services.

## Data availability statement

The data that support the findings of this study are available from the Institute of Population Research of Peking University (IPR). Restrictions apply to the availability of these data, which were used under license for this study. Data are available with the permission of IPR. Requests to access these datasets should be directed to 00-86-010-62751974.

## Ethics statement

Written informed consent was obtained from the individual(s) for the publication of any potentially identifiable images or data included in this article.

## Author contributions

Y-RW is responsible for conceptualizing, data analysis, formal interpretation of results, and writing the manuscript. Y-NL is responsible for conceptualizing, critical revision of article for important intellectual content, and writing—review and editing. Y-LF and Y-YF are responsibility for revision of article. X-YZ is responsible for conceptualizing the study, methodology, and supervision. All authors have read and agreed to the published version of the manuscript.
